# A new estimation of protein-level false discovery rate

**DOI:** 10.1186/s12864-018-4923-3

**Published:** 2018-08-13

**Authors:** Guanying Wu, Xiang Wan, Baohua Xu

**Affiliations:** 1 The Dental Center of China-Japan Friendship Hospital, Beijing, China; 2 ShenZhen Research Institute of Big Data, ShenZhen, China

**Keywords:** FDR, Proteomics, Permutation, Null distribution

## Abstract

**Background:**

In mass spectrometry-based proteomics, protein identification is an essential task. Evaluating the statistical significance of the protein identification result is critical to the success of proteomics studies. Controlling the false discovery rate (FDR) is the most common method for assuring the overall quality of the set of identifications. Existing FDR estimation methods either rely on specific assumptions or rely on the two-stage calculation process of first estimating the error rates at the peptide-level, and then combining them somehow at the protein-level. We propose to estimate the FDR in a non-parametric way with less assumptions and to avoid the two-stage calculation process.

**Results:**

We propose a new protein-level FDR estimation framework. The framework contains two major components: the Permutation+BH (Benjamini–Hochberg) FDR estimation method and the logistic regression-based null inference method. In Permutation+BH, the null distribution of a sample is generated by searching data against a large number of permuted random protein database and therefore does not rely on specific assumptions. Then, *p*-values of proteins are calculated from the null distribution and the BH procedure is applied to the *p*-values to achieve the relationship of the FDR and the number of protein identifications. The Permutation+BH method generates the null distribution by the permutation method, which is inefficient for online identification. The logistic regression model is proposed to infer the null distribution of a new sample based on existing null distributions obtained from the Permutation+BH method.

**Conclusions:**

In our experiment based on three public available datasets, our Permutation+BH method achieves consistently better performance than MAYU, which is chosen as the benchmark FDR calculation method for this study. The null distribution inference result shows that the logistic regression model achieves a reasonable result both in the shape of the null distribution and the corresponding FDR estimation result.

## Background

In shotgun proteomics, the identification of proteins is a two-stage process: peptide identification and protein inference [1]. In peptide identification, experimental MS/MS spectra are searched against a sequence database to obtain a set of peptide-spectrum matches (PSMs) [2–4]. In protein inference, individual PSMs are assembled to infer the identity of proteins present in the sample [5–7].

Inferred proteins are the most biologically relevant outcome of a shotgun experiment. Therefore, the ability of accurately inferring proteins and directly assessing such inference results is critical to the success of proteomics studies. To date, many effective protein inference algorithms have been developed such as ProteinProphet, ComByne and MSBayesPro. However, the problem of accurate assessment of statistical significance of protein identifications remains an open question [8, 9]. Past research efforts towards this direction can be classified into *p*-value based approaches and false discovery rate (FDR) approaches:

- *p*-value based approaches provide a single protein-level *p*-value for each reported protein.
- FDR approaches apply a single threshold to all proteins identified from the data rather than generate individual significance values for each protein.

Both *p*-value based approaches and FDR approaches aim at controlling the quality of identified proteins, though they consider this problem from different perspectives. Unfortunately, available methods still deserve certain drawbacks, as summarized below:

1. **Reliance on specific assumptions.** Most methods depend on particular assumptions regarding the model or the distribution of false positive matches. For instance, the *p*-value based PROT_PROBE approach [10] assumes that protein identification by a collection of spectra follows a binomial model. Similarly, the representative of FDR approaches, MAYU [11] is based on the assumption that false positive PSMs are equally likely to map to either the target or decoy database and the number of false positive protein identifications is assumed to be hypergeometrically distributed.
2. **Reliance on the two-stage calculation process.** Generally, the protein-level confidence measure is obtained by combining peptide-level *p*-values (e.g., [8]). Such process may propagate errors at the peptide-level to the protein-level in a non-trivial manner [12].

Based on above observations, we propose a new framework for the protein-level FDR estimation that can avoid above-mentioned shortcomings. In this framework, we are permuting protein sequences and performing searching against these fake sequences on a dataset to get the corresponding null distribution at the protein-level before *p*-value and FDR calculation. Therefore, our calculation does not rely on the two-stage calculation process. In addition, we do not need to make any assumption on the distribution of protein identification scores since the permutation procedure is non-parametric. More importantly, once the null/permutation distribution is available, we can calculate *p*-values and the FDR without searching a decoy database. Experimental results on several real proteomics datasets show that our framework is effective in *p*-value and FDR calculation and outperforms MAYU consistently.

Although this framework is very appealing, the time required to perform the permutation procedure renders it infeasible to generate the null in an on-line manner before we have a fast permutation algorithm. To alleviate this problem, we suggest to do the permutation in an off-line manner and then store the null distributions for future use. When null distributions built on existing samples are not applicable in analyzing new-coming data with different features, we propose to use logistic regression to infer the null distribution from existing null distributions.

The rest of paper is organized as follows: “Methods” section illustrates the details of our methods. “Results and discussion” section presents the experiment results. “Discussion” section concludes the paper.

## Methods

### Overview of the methods

Proteins that are present in an experimental sample are true positives; others are false positives. Each protein is associated with a score measuring its confidence. The higher the score, more confident we are that the protein is in the sample. If we treat the protein score as a test statistic, the distribution formed by scores of false positives is the null distribution. Given *N* proteins, we can determine the *p*-value of each protein from the null distribution. For a certain FDR *α*, we can determine how many proteins are accepted based on their *p*-values according to the Benjamini-Hochberg (BH) method:


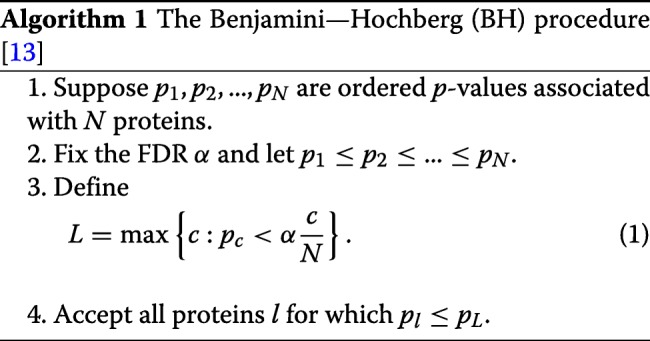


Given a subset of proteins obtained by setting a protein score threshold, we can also determine the FDR according to the BH procedure.

In the method, the *p*-value calculation method and the BH procedure are well-established statistical routines. The major source of errors in estimating the FDR may come from the null distribution estimation. Generally, the more data we have, the more accurate the null distribution. Thus, we estimate the null distribution by using a permutation method, which can generate plenty of data for robust data analysis. However, the permutation method is inefficient. This method becomes computational expensive when handing large datasets. This motivates us to develop a method that can infer the null distribution of a new dataset from null distributions of known datasets. We can store the previous estimated null distributions and conduct the protein-level FDR estimation in an off-line mode. In this way, both accuracy and efficiency can be achieved. Details are provided in the following sections.

### The permutation method

We employ the target-decoy technique to determine null distributions. In the permutation step, each sequence in the original protein database is randomly shuffled [14]. The shuffled proteins are appended into the original protein database to form a concatenated database. Then, proteins are identified from the concatenated target-decoy database. Protein identifications mapping to decoy sequences are false positives, whose scores are used to form the null distribution. When the sample size is small, we may not have enough false positives to form a reliable null distribution. Thus, the shuffling step and the protein inference step are repeated multiple times (e.g. 20 repeats). In each iteration, decoy protein scores are stored.

Suppose we obtain *M* decoy proteins in the above step. Let $\mathcal {Z}=\{z_{1}, z_{2},...,z_{l},...,z_{M}\}$ be the set of decoy protein scores. We partition the range of *z*_*l*_ values into *K* bins of equal length:

1 $$ \mathcal{Z}=\bigcup_{k=1}^{K}{\mathcal{Z}_{k}}.  $$

Here, $\mathcal {Z}_{k}$ contains protein scores belonging to the *k*-th bin. Define *y*_*k*_ as the count in the *k*-th bin:

2 $$ y_{k}=\#\{z_{s}\in \mathcal{Z}_{k}\}.  $$

and let *x*_*k*_ be the center point of $\mathcal {Z}_{k}$. Note that $\sum _{i=1}^{K}{y_{k}}=M$. Then, the set of points $\mathcal {H}=\{(x_{1},y_{1}/M),...,(x_{K},y_{K}/M)\}$ describes the probability density function of the null distribution.

When a protein belongs to $\mathcal {Z}_{\hat {k}},\hat {k}=1,2,...K$. Then, its *p*-value can be approximated as:

3 $$ p_{l,z_{l}\in \mathcal{Z}_{\hat{k}}}=\frac{\sum_{k=\hat{k}}^{K}{y_{k}}}{M}.  $$

Given *N* proteins, we can estimate their *p*-values. The determination of the relationship of the FDR and the number of proteins is straightforward by applying the BH procedure mentioned in the previous section.

In the permutation method, we need to shuffle and identify proteins multiple times. Thus, the notable limitation of the permutation method is its low efficiency. Null distributions of different samples can be stored in the protein database for future use in an off-line mode.

### A general null distribution inference model

The protein identification result can be affected by various reasons such as the tandem MS peak count and the sample complexity. Null distributions built on existing samples may not be applicable in analyzing data with different tandem MS peak counts and a different sample complexity. Determining the null distribution of new data is time consuming by applying the permutation method. Thus, we design a way to infer the null distribution from existing null distributions in the case that high efficiency is desired.

A raw data can be described by many features. For instance, tandem MS peak counts and tandem MS spectral quality measured by the mean noise level. Suppose we have *I* existing samples and each sample can be described by *J* features. Denote features of the *i*-th sample as (*r*_*i*,1_,*r*_*i*,2_,...,*r*_*i*,*J*_) and let $\mathcal {H}_{i}(x)$ be the null probability density function associated with the sample. The feature of a new sample is denoted as (*r*_0,1_,*r*_0,2_,...,*r*_0,*J*_) and our objective is to infer its null density function $\mathcal {H}_{0}(x)$.

For a protein score belonging to the *k*-th bin $\tilde {x}\in \mathcal {Z}_{k}$, we collect the following information from existing samples: Then, the relationship of the probability $\text {Pr}_{i,k}(x\leq \tilde {x}|\tilde {x}\in \mathcal {Z}_{k})$ and *J* features can be described by the following logistic regression model:

4 $$ \log\left(\frac{\text{Pr}_{i,k}}{1-\text{Pr}_{i,k}}\right)=\beta_{k,0} + \sum_{j=1}^{J}{\beta_{k,j}r_{i,j}},i=1,2,...,I.  $$

After fitting the logistic regression model, we estimate the the probability $\text {Pr}_{0}(x\leq \tilde {x}|\tilde {x}\in \mathcal {Z}_{k})$ of the new sample as:

5 $$ \text{Pr}_{0}(x\leq \tilde{x}|\tilde{x}\in \mathcal{Z}_{k})=\frac{1}{1+e^{-\left(\beta_{k,0} + \sum_{j=1}^{J}{\beta_{k,j}r_{0,j}}\right)}}.  $$

For bins $\mathcal {Z}_{1}, \mathcal {Z}_{2}$,... and $\mathcal {Z}_{K-1}$, we collect information as shown in Table 1, conduct logistic regression by model (4) and obtain the fitting coefficients *β*_*k*,*j*_(*k*=1,2,...*K*−1;*j*=0,1,2,...,*J*) as shown in Table 2. It is unnecessary to perform logistic regression on the last bin $\mathcal {Z}_{k}$ because $\text {Pr}_{i}(x\leq \tilde {x}|\in \mathcal {Z}_{K})=1,i=0,1,2,...,I$. We use a coefficient table to store the information:

**Table 1:** The probability $\text {Pr}_{i,k}(x\leq \tilde {x}|\tilde {x}\in \mathcal {Z}_{k})$ can be calculated from the null probability density function $\mathcal {H}_{i}(x)$

Sample	feature 1	...	feature *j*	...	feature *J*	$\text {Pr}_{i}(x\leq \tilde {x}|\tilde {x}\in \mathcal {Z}_{k})$
Sample 1	*r*_1,1_	...	*r*_1, *j*_	...	*r*_1, *J*_	*P*_1, *k*_
...	...	...	...	...	...	...
Sample *i*	*r*_*i*,1_	...	*r*_*i*, *j*_	...	*r*_*i*, *J*_	*P*_*i*, *k*_
...	...	...	...	...	...	...
Sample *I*	*r*_*I*,1_	...	*r*_*I*, *j*_	...	*r*_*I*, *J*_	*P*_*I*, *k*_

**Table 2:** The coefficient table for null distribution inference

	Intercept	feature 1	feature 2	...	feature J
1-th bin	*β*_1,0_	*β*_1,1_	*β*_1,2_	...	*β*_1, *J*_
...	...	...	...	...	...
*k*-th bin	*β*_*k*,0_	*β*_*k*,1_	*β*_*k*,2_	...	*β*_*k*, *J*_
...	...	...	...	...	...
(*K*−1)-th bin	*β*_*K*−1,0_	*β*_*K*−1,1_	*β*_*K*−1,2_	...	*β*_*K*−1, *J*_

Then, the density function $\mathcal {H}_{0}(x)$ can be approximated as:

6 $$ \mathcal{H}_{0}(x\in \mathcal{Z}_{k})=\text{Pr}_{0}(x\leq \tilde{x}|\tilde{x}\in \mathcal{Z}_{k}) - \text{Pr}_{0}(x\leq \tilde{x}|\tilde{x}\in \mathcal{Z}_{k-1}).  $$

When *k*=1, $\mathcal {H}_{0}(x\in \mathcal {Z}_{k})$ becomes ill-posed because $\mathcal {Z}_{0}$ is undefined. In this case, let $\text {Pr}_{0}(x\leq \tilde {x}|\tilde {x}\in \mathcal {Z}_{0})=0$. By using the coefficient table and equation (6), we obtain the null density function $\mathcal {H}_{0}$.

### The feature protein database

**Fig. 1:**
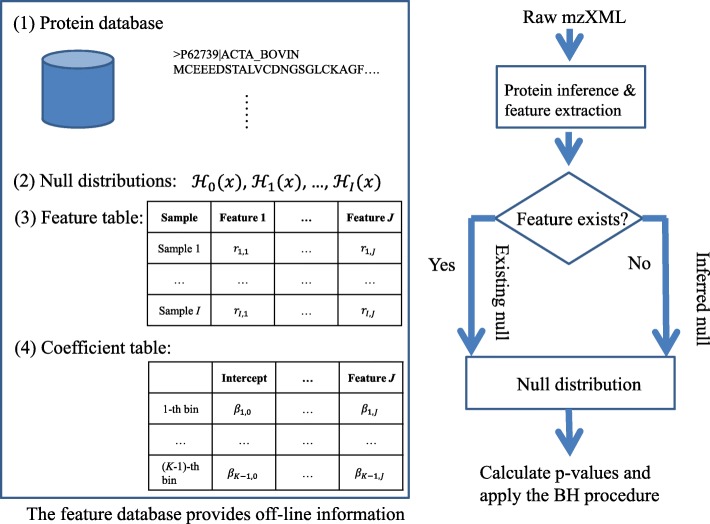
A feature database contains null distributions, a feature table and a coefficient table. When features of a new sample exist in the feature table, the corresponding null distribution is chosen. Otherwise, a null distribution is inferred with information in the coefficient table. Then the null distribution is used to perform the protein-level FDR estimation

We can use a feature table to organize our data. A feature database is shown in Fig. 1. The feature database contains a protein database, which is used to perform protein inference. The null distributions in the feature database are obtained by the permutation method based on existing samples. For each existing sample, its features are extracted and stored in the feature table. The logistic regression coefficients are obtained by fitting the model (4) on the existing samples. The off-line strategy means that: To obtain the null distribution of a new sample, we neither need to apply the permutation method nor have to perform logistic regression fitting.

When a new raw data is input, protein inference and feature extraction are performed. We can use existing protein inference algorithms such as ProteinProphet to identify proteins from the protein database in the feature database. Then, we can compare the new sample with samples in the feature database based on their features. We can measure the similarity of two samples by calculating the correlation of their feature vectors. Similar samples are often encountered when we analyze replicate samples. If the similarity between the new sample and an existing sample *i* is high (e.g. the correlation of features is above 0.9), we use $\mathcal {H}_{i}(x)$ as the null distribution to calculate the protein-level FDR. If we cannot find any similar sample in the feature database, we can plug the coefficients in the coefficient table into function (5) and use Eq. (6) to infer a new null distribution for the new sample.

The permutation method takes lots of time. When the number of bins in the null distribution and the number of features are large, the logistic regression fitting may also take a great amount of time. The off-line information (i.e. the feature table and the coefficient table) is used to achieve a new null distribution without the permutation step and the logistic regression fitting step. Thus, it makes the protein-level FDR estimation efficient.

### Our current implementation of the framework

When applying the proposed protein-level FDR estimation framework, two key points are: features and similarity measurement. A sample can be described by features. When a novel sample is similar to an existing sample by comparing their features, the null distribution of the existing sample will be used. Otherwise, a new null distribution is inferred by applying the logistic model based on sample features.

The error propagation from the peptide-level to the protein-level is non-trivial. Features of raw data such as tandem MS peak counts are faraway from the final protein inference result. Thus, these kinds of features may not have a clear connection with protein scores. In our current implementation, we determine to directly select features from protein scores.

First, we partition the range of protein scores of sample *i* into 10 bins of equal length. The probabilities of protein scores falling in 10 bins are denoted as *P*_*i*,1_, *P*_*i*,2_,...,*P*_*i*,10_. Then, we choose sample *j* as a reference sample. The similarity of protein identification results of sample *i* and sample *j* is measured by their Kullback-Leibler (KL) divergence:

7 $$ D_{i,j}=\sum_{k=1}^{10}{\text{Pr}_{i,k}\log{\frac{\text{Pr}_{i,k}}{\text{Pr}_{j,k}}}}.  $$

The smaller the value of *D*_*i*,*j*_, the more similar sample *i* and sample *j*. We choose the KL divergence from each sample to the reference sample as a feature, which is used to infer the null distribution and measure the sample similarity.

### Overview of the experiments

The whole framework consists of two parts: The first part employs the permutation method and the BH procedure to estimate the FDR (Permutation+BH); the second part provides a logistic regression model to infer the null distribution of a new sample based on existing null distributions. We first conduct the experiment to verify Permutation+BH in FDR estimation. The performance of our method is compared to MAYU based on three datasets with groundtruth. Then, we conduct another experiment to illustrate the performance of our null distribution inference method. In the last part of our experiments, we discuss the reference dataset issue in our current implementation of our framework.

The whole framework is implemented in Ruby (v1.9.2p290). Target-decoy concatenated databases are generated from UniProtKB/Swiss-Prot (Release 2011_01) by appending shuffled protein sequences into the original protein database. Peptides are identified by X!Tandem (v2010.10.01.1) [4]. Then, ProteinProphet (Embedded in the Trans-Proteomic Pipeline v4.5 RAPTURE rev 0, Build 201109211427) is employed to perform peptide probability calculation and protein inference, respectively [5, 15].

In our experiments, we use six public available datasets: ISB, ABRF, Yeast, Yeast_Train, Human and Human_Test. The ISB dataset was achieved from a 18 standard protein mixture [16]. The sample was analyzed on a Waters/Micromass Q-TOF using an electrospray source. The ABRF sPRG2006 dataset contains 49 standard proteins. The Yeast and the Yeast_Train dataset were obtained by analyzing cell lysate on both LCQ and ORBI mass spectrometers from wild-type yeast grown in rich medium [17, 18]. The dataset contains a protein reference set which is used as the groundtruth. The Human dataset was obtained from human HEK293T cell lines and analyzed on the ORBI mass spectrometer. The Human_Test dataset was obtained by analyzing human serum samples with Thermo LTQ-FT. In our experiments, “.RAW” files are converted to “.mzXML” files by TPP. The addresses to access these datasets are shown in Table 3:

**Table 3:** Names and URLs of data files

Dataset name	File name	URL
ISB	QT20051230_S_18mix_04.mzXML	http://regis-web.systemsbiology.net/PublicDatasets/
ABRF	Lane/060121Yrasprg051025ct5.RAW	https://proteomecommons.org/dataset.jsp?i=71610
Yeast	YPD_ORBI/061220.zl.mudpit0.1.1/raw/000.RAW	http://aug.csres.utexas.edu/msnet/
Yeast_Train	YPD_ORBI/070119-zl-mudpit07-1/raw/000.RAW	http://aug.csres.utexas.edu/msnet/
Human	YPD_LCQ/060b.RAW	http://aug.csres.utexas.edu/msnet/
Human_Test	PAe000281_mzXML_200909301914/B06-7017_c.mzXML	http://www.peptideatlas.org/repository/

## Results and discussion

### FDR estimation

In this experiment, the first three datasets are used: ISB, ABRF and Yeast. For the ISB dataset, the 18 standard proteins together with 15 contaminants are marked as the groundtruth [16]. For the ABRF dataset, the 49 standard proteins and 78 contaminants are used as the groundtruth. Readers can refer to the supplementary document for more information [19]. For the Yeast dataset, all proteins in the protein reference set are treated as true proteins.

The permutation method includes two steps to obtain a null distribution: a shuffling step and a protein inference step. In the shuffling step, each protein sequence is shuffled and appended into the original protein database. In the protein inference step, proteins are identified from the target-decoy concatenated database with TPP. The shuffling step and the protein inference step repeat for 20 times. The protein mapping to a decoy sequence is considered to be a false positive. The protein probabilities of all false positives from the 20 protein identification results are collected. Then, the histogram of the protein probabilities is built and used as the null probability density function. In general, the smaller the bin size, the more detail the null distribution contains and more data points are desired to build the null distribution. In our experiments, the length of each bin is empirically chosen to be 0.003.

After the null distribution has been built, the *p*-value of each protein is calculated according to Eq. (3). Next, *p*-values of all proteins are sorted in the ascending order. Then, the BH procedure is conducted on *p*-values to obtain the relationship of the number of proteins and the FDR.

**Fig. 2:**
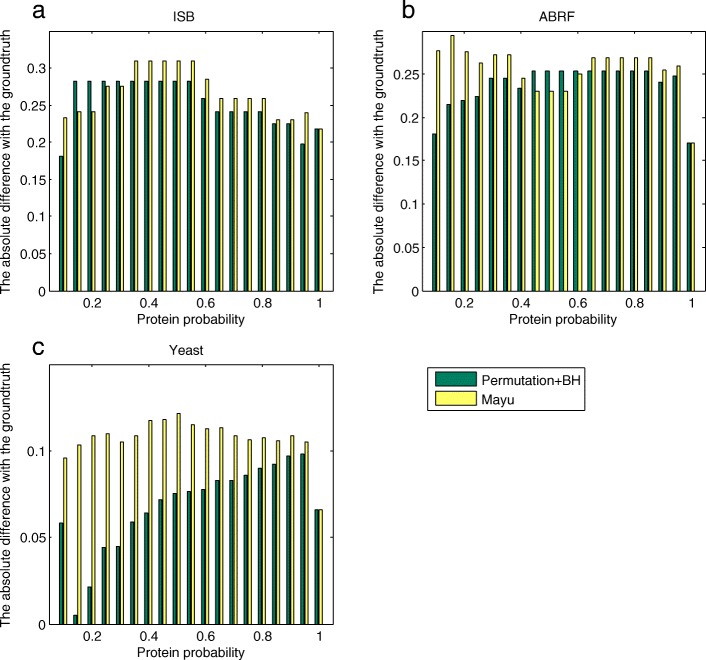
**a**, **b** and **c** show the absolute differences between FDRs estimated and true FDRs. The smaller the difference, the better the performance. In (**a**), our method is comparable with MAYU. In (**b**), our method is better than MAYU on average. In (**c**), our method is dominantly better than MAYU

We apply our method and MAYU to the three protein datasets to estimate the FDR. The true FDR is calculated as the ratio of the number of proteins belonging to the groundtruth set and the total number of protein identifications. The performances of different methods are validated by comparing the absolution difference between the estimation and the groundtruth. The results based on three datasets are shown in Fig. 2.

According to our experimental results, our method and MAYU are comparable in performance on the ISB dataset. For the ABRF dataset, our method is better than MAYU on average. Our method is dominantly better than MAYU on the Yeast dataset.

### Null distribution inference

In this dataset, the first five datasets listed in Table 3 are used to fit the logistic regression model (4) and the Human_Test dataset is used to validate the null distribution inference result.

In this experiment, we choose the ISB dataset as a reference dataset and calculate the KL divergence from other samples to the ISB dataset by using Eq. (7). The feature table for six datasets is shown in Table 4.

**Table 4:** The feature table for five datasets and one test set

Sample	KL divergence
ISB	0.000
ABRF	0.0301
Yeast	0.3697
Human	0.2206
Yeast_Train	0.2781
Human_Test	0.4159

The null distributions of six samples are obtained by the permutation method. The first five null distributions are used to infer the null distribution of Human_Test. The last null distribution will be used as a reference.

Let the first five samples be sample 1,2,3,4 and 5, respectively. Human_Test is denoted as sample 0. The KL divergence from sample *i* to the reference sample is denoted as *r*_*i*,1_. Since the bin length 0.003, the number of bins is *K*=334. The probability $\text {Pr}_{i}(x\leq \tilde {x}|\tilde {x}\in \mathcal {Z}_{k})$ is calculated from the null distribution of sample *i* for bin *k*. The logistic regression coefficients for bin *k* are obtained through the following model:

8 $$  \min_{\beta_{k,0},\beta_{k,1}}\sum_{i=1}^{5}{\mathcal{L}\left(\log\left(\frac{\text{Pr}_{i,k}}{1-\text{Pr}_{i,k}}\right)-\beta_{k,0} - \beta_{k,1}r_{i,1}\right)}.  $$

Here, *β*_*k*,0_ is the intercept coefficient; *β*_*k*,1_ is the sample KL divergence coefficient, respectively; $\mathcal {L}$ is a loss function measuring the error in estimation. In our experiment, we choose $\mathcal {L}=\big |\big |\cdot \big |\big |_{2}$. In our Ruby program, we implement a R (v2.13.1) interface from which users can call any robust loss function such as the Huber loss. We partially show the coefficient table in Table 5.

**Table 5:** Coefficient table. Note that we only need to conduct logistic regression on the first *K*−1 bins

Bins	Intercept coefficients	KL divergence coefficients
1-th bin	-6.0539	-18.6072
...	...	...
333-th bin	5.1731	3.3703

**Fig. 3:**
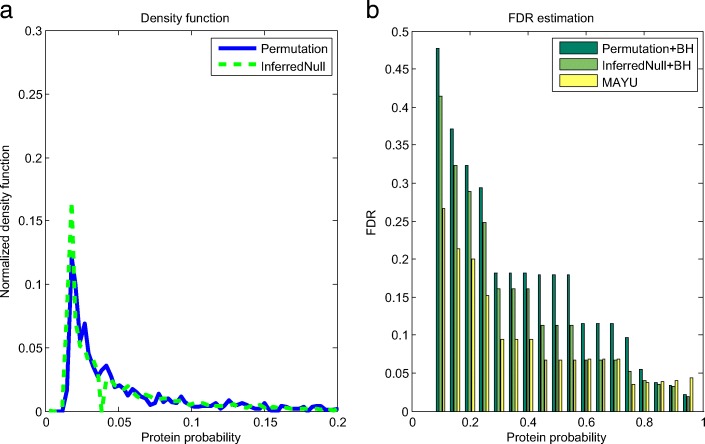
**a** shows the probability density functions of the inferred null distribution and the null distribution obtained by the permutation method. The correlation between the inferred null distribution and the generated null distribution using permutation is 0.9052. **b** shows the FDR estimation results of Permutation+BH, InferredNull+BH and MAYU

The feature table and the coefficient table are stored in the feature database. When analyzing the new sample (i.e. Human_Test), we just plug coefficients in the coefficient table in Eq. (5) and use Eq. (6) to get the null probability density function of Human_Test. The result is shown in Fig. 3.

Figure 3a shows the probability density functions of the inferred null and the null obtained by the permutation method. The peak height of the inferred null is overestimated compared with that of the permutation null. In Fig. 3b, Permutation+BH and InferredNull+BH estimate the FDR by applying the BH procedure to the null distribution generated by the permutation method and the inferred null distribution, respectively. According to the result shown in Fig. 3b, the performance of InferredNull+BH is closer to Permutation+BH than that of MAYU. The correlation of the inferred null distribution and the permutation generated null distribution is 0.9052.

### The reference dataset

**Fig. 4:**
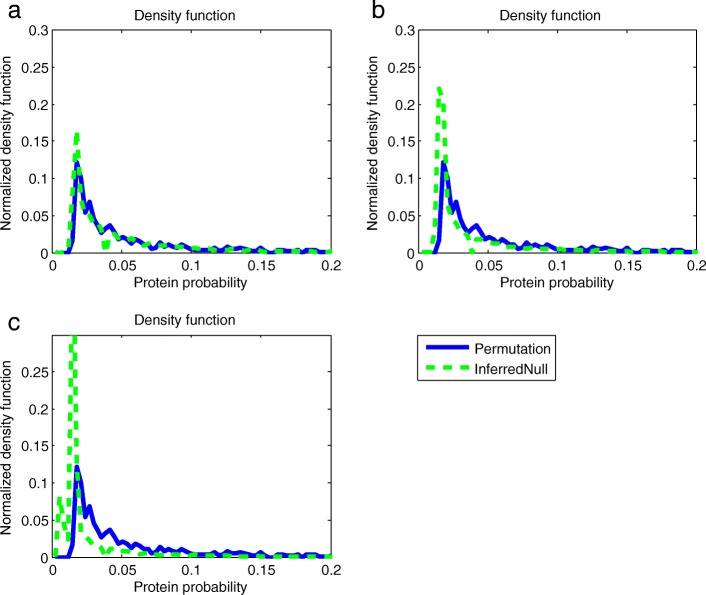
**a**, **b** and **c** show null distribution inference results by using the ISB dataset, the ABRF dataset and the Yeast dataset as a reference dataset, respectively. The correlations between the inferred null distribution and the permutation generated null distribution in (**a**), (**b**) and (**c**) are 0.9052, 0.6779 and 0.3317, respectively

In our current implementation of our framework, we need to reference dataset. The reference dataset is chosen to calculate the KL divergence of each sample to the reference dataset. Then, the KL divergence is used as a feature in both similarity measurement and null distribution inference. In the previous experiment, we take the ISB dataset as the reference dataset. The KL divergence is a non-symmetric measure of the difference between two probabilities. Thus, a different reference dataset may lead to a different null distribution inference result. In the following experiment, we take the ISB dataset, the ABRF dataset and the Yeast dataset as the reference dataset, respectively. The result is shown in Fig. 4. The correlations of the inferred null distribution and the permutation generated null distribution when using the ISB dataset, the ABRF dataset and the Yeast dataset as a reference dataset are 0.9052, 0.6779 and 0.3317, respectively. According to the experimental result, the best performance in null distribution estimation is achieved when the ISB dataset, which contains 18 proteins, is taken as the reference dataset. The performance is worst when the Yeast dataset containing hundreds of proteins is used as the reference dataset.

**Fig. 5:**
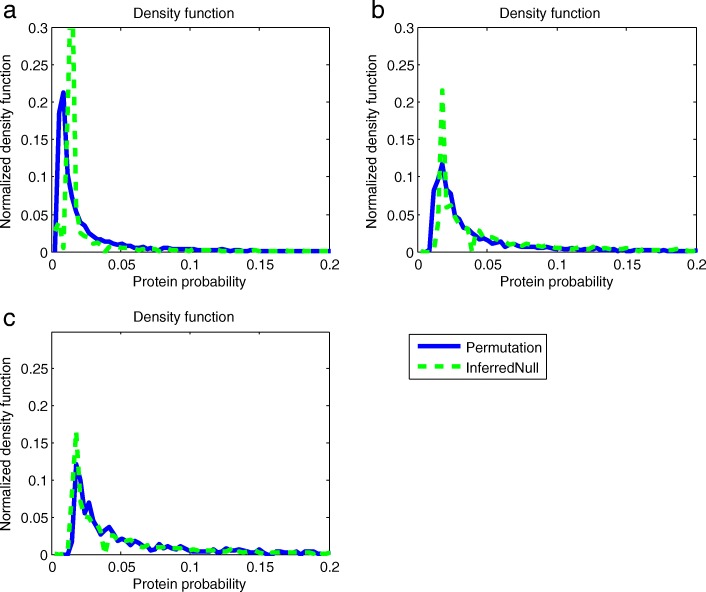
In (**a**), the ABRF dataset is used as the test data and other datasets are the training data; in (**b**), the Yeast dataset is used as the test data and the other datasets are training data; in (**c**), the Human_Test dataset is used as test data and other datasets are the training data. The correlations between the inferred null distribution and the permutation generated null distribution in (**a**), (**b**) and (**c**) are 0.4259, 0.8116 and 0.9052, respectively

Readers may be interested in the results of inferring the null distributions of other samples (other than Human_Test) by using the ISB dataset as the reference dataset. In the following experiment, we conduct two extra experiments to inferring the null distributions of the ABRF dataset and the Yeast dataset. In each of the two experiments, we treat either the ABRF dataset or the Yeast dataset as test data and using remaining datasets as training data. The result is shown in Fig. 5.

The difference between the inferred null distribution and the empirical null distribution obtained by the permutation method may be caused by the following reasons:

- The data used in fitting the logistic regression model may be neither typical nor enough. Data representative to different conditions are desired to obtain a robust regression model. When information of some typical conditions are missing, it is may be hard to make it up by using mathematical models.
- In our experiment, we only consider one feature in our logistic model. The single feature may not explain all kinds of variation in the protein inference process. A more accurate model can be achieved by using more features and plenty of data in building the feature database.
- Even if we obtain an ideal logistical model with perfect coefficients, it is not guaranteed that the new data is not an outlier. It is often the case that the new data locates at a point that has a certain distance to the ideal model.

The biggest advantage of the null inference method is its efficiency. Once the coefficient table is obtained beforehand, the inference of the null distribution is just the calculation of deterministic functions (5) and (6). The whole process of the FDR estimation just takes a few seconds. This should benefit the large-scale data analysis.

## Discussion

In the Permutation+BH method, we use the permutation method to generate the null distribution and apply the BH procedure to estimate the FDR. The method does not relies on specific assumptions and works directly at the protein-level. Thus, the problems related to improper assumption and error propagation are avoided. The flexibility of our method also implies that it can be used with any search method or protein inference method of the user’s choice. According to our experimental results based on three datasets, our method performs better than MAYU. We believe that this is partly due to a more accurate estimation of the null distribution through the increased sampling by our permutation method, than in a typical 1:1 target-decoy approach as used in MAYU.

In the Permutation+BH method, the efficiency is low because we need to shuffle protein sequences and conduct protein inference multiple times. We propose an off-line strategy to handle this issue. In the off-line strategy, a feature protein database is built beforehand with null distributions obtained from existing samples, a feature table and a coefficient table. When a new sample cannot find a match in the feature table, a new null distribution is inferred by directly plugging the coefficients in the coefficient table into the logistic model. The logistic regression model provides an efficient way to infer the null distribution. Our model is currently trained only on a few samples and including only 1 feature, limiting its accuracy. We will seek to improve this model by adding many more features and training the model on more datasets, as part of our future work.

## Conclusions

In this paper, we propose a protein-level FDR estimation framework. The framework includes two major components: the Permutation+BH FDR estimation method and the logistic regression-based null distribution inference method. The Permutation+BH method first applies the permutation to generate the null distribution and then uses the BH procedure to estimate the FDR. However, this method is inefficient for online identification. Therefore, we propose the logistic regression-based null distribution inference method to handle this issue. In our experiment based on three public available datasets, our Permutation+BH method achieves consistently better performance than MAYU, which is chosen as the benchmark FDR calculation method for this study. The null distribution inference result shows that the logistic regression model achieves a reasonable result both in the shape of the null distribution and the corresponding FDR estimation result.
